# Rapid and reversible lithiation of doped biogenous iron oxide nanoparticles

**DOI:** 10.1038/s41598-019-38540-8

**Published:** 2019-02-12

**Authors:** Masaaki Misawa, Hideki Hashimoto, Rajiv K. Kalia, Syuji Matsumoto, Aiichiro Nakano, Fuyuki Shimojo, Jun Takada, Subodh Tiwari, Kenji Tsuruta, Priya Vashishta

**Affiliations:** 1 Faculty of Science and Engineering, Kyushu Sangyo University, Fukuoka, 813-8503 Japan; 2 Department of Physics, Kumamoto University, Kumamoto, 860-8555 Japan; 3 Collaboratory for Advanced Computing and Simulations, Department of Physics & Astronomy, Department of Computer Science, Department of Chemical Engineering & Materials Science, and Department of Biological Sciences, University of Southern California, Los Angeles, CA 90089-0242 USA; 4 Department of Applied Chemistry, School of Advanced Engineering, Kogakuin University, Tokyo, 192-0015 Japan; 5 Core Research for Evolutionary Science and Technology (CREST), Japan Science and Technology Agency (JST), Okayama University, Okayama, 700-8530 Japan; 6 Graduate School of Natural Science and Technology, Okayama University, Okayama, 700-8530 Japan

## Abstract

Certain bacteria produce iron oxide material assembled with nanoparticles (NPs) that are doped with silicon (Fe:Si ~ 3:1) in ambient environment. Such biogenous iron oxides (BIOX) proved to be an excellent electrode material for lithium-ion batteries, but underlying atomistic mechanisms remain elusive. Here, quantum molecular dynamics simulations, combined with biomimetic synthesis and characterization, show rapid charging and discharging of NP within 100 fs, with associated surface lithiation and delithiation, respectively. The rapid electric response of NP is due to the large fraction of surface atoms. Furthermore, this study reveals an essential role of Si-doping, which reduces the strength of Li-O bonds, thereby achieving more gentle and reversible lithiation culminating in enhanced cyclability of batteries. Combined with recent developments in bio-doping technologies, such fundamental understanding may lead to energy-efficient and environment-friendly synthesis of a wide variety of doped BIOX materials with customized properties.

## Introduction

Biologically produced materials exhibit amazing properties that often surpass those of the best man-made materials^1^. For example, microbes such as bacteria have remarkable abilities to synthesize materials with intricate nanostructures with outstanding properties^2–5^. Such biomineralization played crucial roles in the geological history of the Earth^3^. Since it usually occurs under ambient conditions, biomineralization is also utilized in environment-friendly renewable energy technologies such as the production of acetate from carbon oxide and sunlight^4^. An archetypal example of biomineralization is provided by iron (Fe)-oxidizing bacteria, which use divalent iron (Fe^2+^) in aqueous environment as an energy source^2,6–9^. These bacteria are ubiquitous since Fe is the fourth most abundant element in the Earth’s crust^10^. Their metabolism oxidizes Fe^2+^ into Fe^3+^ to gain energy, while depositing the product of the chemical reaction as iron oxide (Fe_2_O_3_) solid. Despite a hundred years of recognition of their importance^11^, however, detailed knowledge about their physiology, chemistry and mechanics is severely limited. Aquatic iron-oxidizing bacteria, *Leptothrix ochracea*, produce Fe^3+^-based amorphous oxide microtubular sheaths consisting of nanoparticles (NPs) of diameter ~2 nm^5,12^. This biogenous iron oxide (BIOX) was found to be amorphous and doped with silicon (Si), with cation composition of Fe:Si ~ 3:1^13,14^. Owing to its unique nanostructure, this material has been studied as a next-generation functional material^15,16^. As a practical matter, nanostructured transition metal-containing mixed oxides, hydroxide, salts and even metal-organic frameworks are widely studied for energy-storage applications^17–32^. Among them, iron-oxide based NPs or nanosheets are promising electrode materials due to its high theoretical capacity, abundance and safety^17–24^. Recently, BIOX was found to exhibit remarkable cyclability when used as an anode in lithium-ion batteries^13,14^. Namely, their Li-storage capacity does not degrade after many lithiation/delithiation cycles, which is an essential property for commercially viable batteries. Since *Leptothrix ochracea* lives worldwide and readily produces BIOX at ambient conditions (*e.g*., the BIOX is widely seen in natural aquatic environment such as swamp or channel as reddish-brown precipitate), it could provide sustainable supply of low-cost, high-performance Li-battery electrode materials in an environment-friendly manner. While the excellent performance of BIOX has been hypothesized to arise from its nanostructure based on 2 nm NP^12^, the microscopic mechanisms underlying the superior cyclability remains elusive. Also not known is the role of the significant amount (~25%) of Si-doping. Thus, the fundamental scientific questions are: (1) how does the BIOX’s nanostructure enhance the cyclability of lithiation, and (2) what is the role of-Si doping?

**Figure 1:**
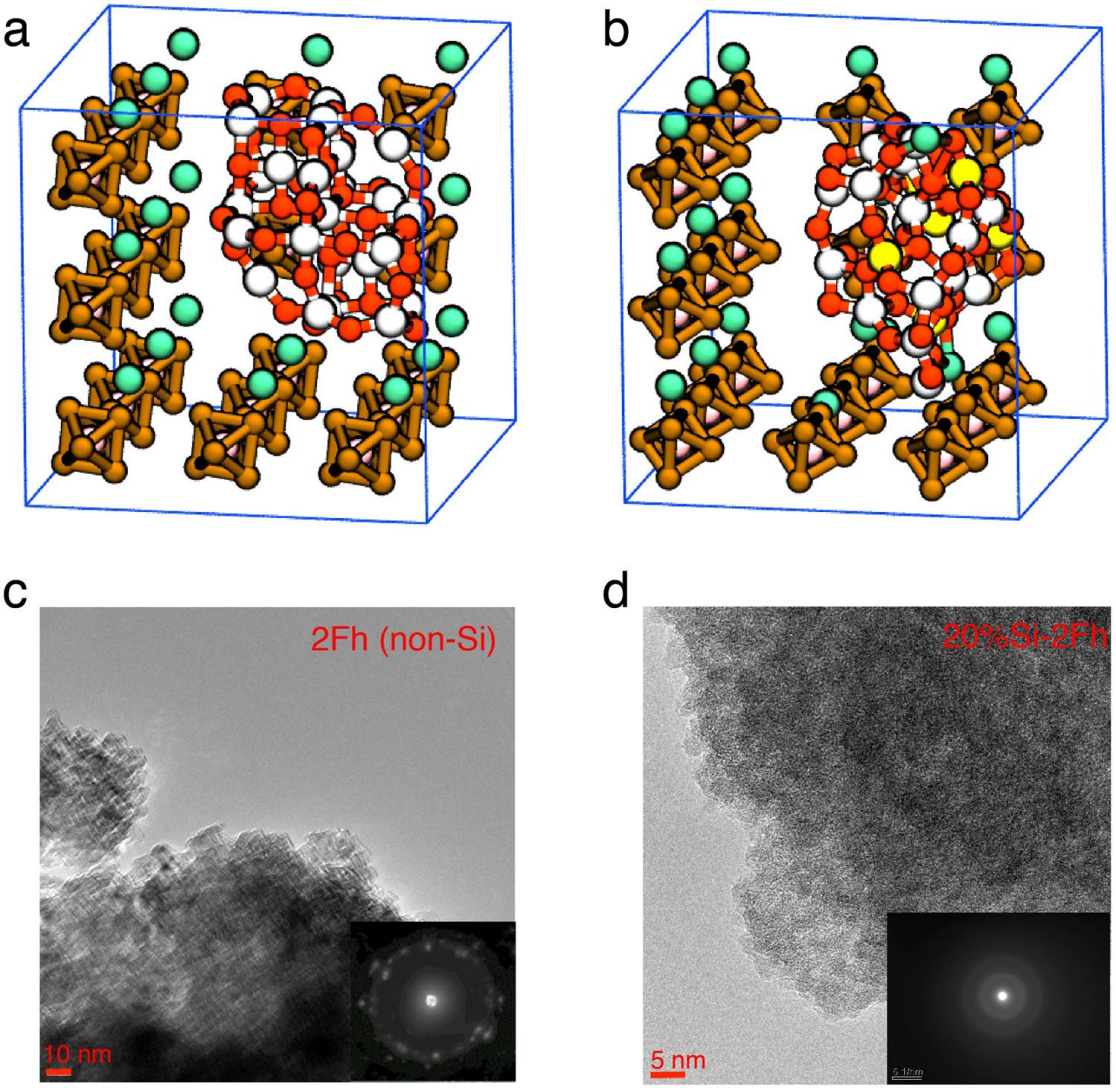
Simulated and synthesized NPs. (**a**,**b**) The initial configurations of the simulation cells with (**a**) Fe_2_O_3_ NP and (**b**) Fe_2_O_3_-SiO_2_ NP in LiPF_6_ electrolyte. White, red, yellow, green, pink, and brown spheres represent Fe, O, Si, Li, P and F, respectively. Blue lines show the cell edges. (**c**,**d)** TEM images of as-synthesized (**c**) 2Fh and (**d**) 20% Si-doped 2Fh, where the insets show ED patterns.

We performed joint simulation and biomimetic-experiment study in order to answer these questions. First principles quantum molecular dynamics (QMD) simulations were performed to study lithiation and delithiation properties of an amorphous Si-free and Si-doped Fe_2_O_3_ NPs, which is immersed in Li^+^(PF_6_)^−^ electrolyte. QMD simulations follow the trajectories of all atoms while computing interatomic forces quantum mechanically from first principles (simulation details are described in methods). We simulated a Si-free NP (Fe_24_O_36_, Fig. 1a) and a Si-doped NP (Fe_18_O_27_-Si_6_O_12_, Fig. 1b), each immersed in 19 LiPF_6_ at a temperature of 500 K. First, the simulation cells were thermalized for 1.33 picoseconds (ps) to confirm the structural stability. Next, 18 electrons were introduced in the simulation cells, and the systems were thermalized for 4.84 ps to study the lithiated state. Subsequently, delithiation dynamics were studied by removing 18 electrons and thermalizing the systems for 1.82 ps.

In order to mirror these QMD simulations, we chemically synthesized biomimetic iron oxide NPs (2-line ferrihydrite, 2Fh) with and without Si-doping (synthesis details are described in methods)^33^. The microstructures of Si-free (Fig. 1c) and Si-doped (Fig. 1d) systems were observed using transmission electron microscopy (TEM). TEM images of Si-doped samples before and after discharging are found in ref.^33^. Both samples consist of primary particles of a diameter of about 5 nm. The electron diffraction (ED) pattern for Si-free sample (inset in Fig. 1c) shows clear spots indicating monophasic crystalline structure of 2Fh, whereas degradation of the spots for the Si-doped sample (inset in Fig. 1d) can be attributed to amorphization of the structure by Si doping. The X-ray diffraction analysis also reveals the inhomogenization dependent on the Si molar ratio (Fig. S1 in supplementary information).

## Surface lithiation

**Figure 2:**
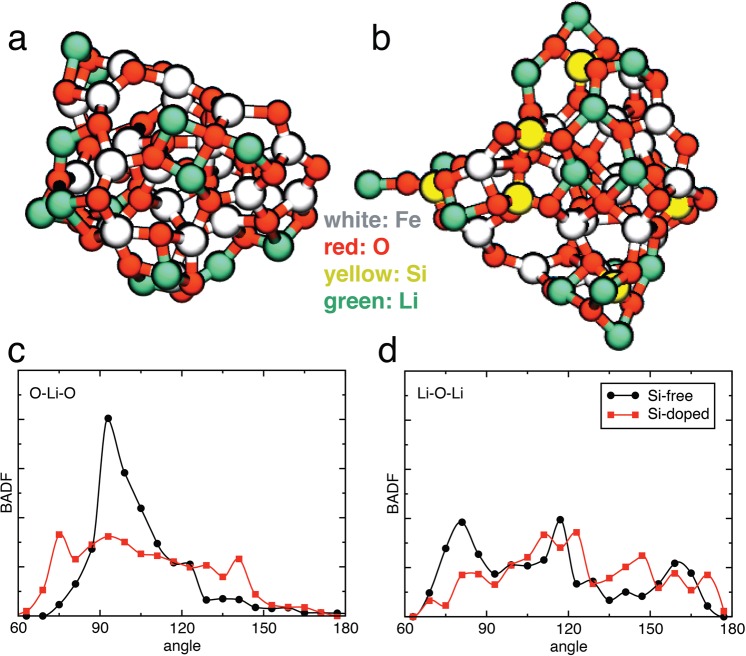
Lithiated NP structure. (**a**,**b**) Snapshots of lithiated (**a**) Si-free and (**b**) Si-doped iron oxide NPs due to charging. White, red, yellow and green spheres represent Fe, O, Si and Li atoms, respectively. (**c**,**d**) Distribution of (**c**) O-Li-O and (**d**) Li-O-Li bond angles in the charged state. The black curve with circles and red curve with squares show the BADFs in the Si-free and Si-doped systems, respectively.

First, we studied lithiated structures due to charging. Figure 2a,b, illustrates the Si-free and Si-doped iron oxide NPs, respectively, with Li atoms adsorbed on the surfaces. In this stage, the numbers of adsorbed Li atoms are the same between the two types of NPs. Poizot *et al*. demonstrated that the lithiation of transition-metal oxide NPs is accompanied by the nucleation of crystalline Li_2_O,

1 $${{\rm{M}}}_{x}{{\rm{O}}}_{y}+2{y}{{\rm{e}}}^{-}+2{y}{{\rm{Li}}}^{+}\to {y}{{\rm{Li}}}_{2}{\rm{O}}+x{\rm{M}}({\rm{M}}={\rm{Fe}},{\rm{Co}},{\rm{Ni}},{\rm{Cu}}),$$

which is distinct from the conventional lithiation mechanism based on Li insertion in bulk materials^34^. On the other hand, it is considered that amorphous Li and Si-based amorphous oxide matrix is formed during the lithiation/delithiation process of BIOX anodes^13^. Figure 2c,d, shows the bond angle distribution functions (BADFs) for O-Li-O and Li-O-Li bonds, respectively. Figure 2c shows a sharp peak in the O-Li-O angle distribution for the Si-free system. This peak corresponds to two-membered (Li-O)_2_ or (Li-O)(Fe-O) rings as seen in Fig. 2a. On the other hand, in the Si-doped system, tetrahedral SiO_4_ units likely hinder the formation of such two-membered rings. This explains the broader O-Li-O angle distribution for the Si-doped system in Fig. 2c. The existence of two-membered rings (as in crystalline Li_2_O) in the Si-free system indicates the formation of crystalline Li_2_O-like fragments in Fe_2_O_3_-NP anodes, which is consistent with previous studies in transition-metal oxide NPs^34^, whereas amorphous Li and Si-based amorphous oxide matrix is formed in BIOX anodes, which again is consistent with previous studies^13^.

## Rapid delithiation

**Figure 3:**
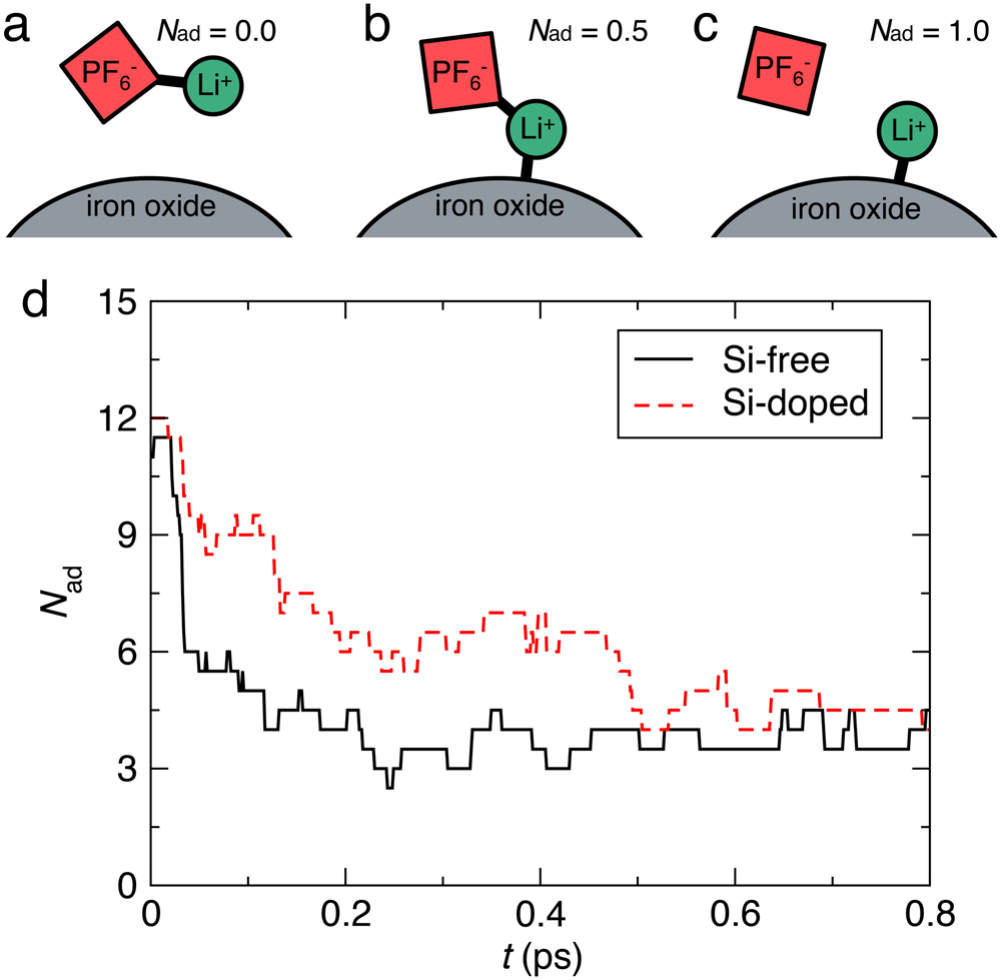
Rapid delithiation of NPs. (**a**–**c**) Three types of the lithiation state: (**a**) delithiated, (**b**) intermediate and (**c**) lithiated states. (**d**) Time evolution of the number of adsorbed Li atoms on the surfaces, after 18 electrons were removed at *t* = 0 ps. The black solid and red dashed curves correspond to the Si-free and Si-doped systems, respectively.

Starting from the lithiated states in Fig. 2, we investigated the delithiation dynamics upon discharging. Figure 3 shows time evolution of the number of Li ions classified by adsorption stages. To investigate the degree of lithiation, we introduce the number of adsorbed Li ions, *N*_ad_: (a) delithiated state, in which Li is not bonded to NP (*N*_ad_ = 0, Fig. 3a); (b) intermediate state, in which Li is bonded to both NP and electrolyte anion (*N*_ad_ = 0.5, Fig. 3b); and (c) lithiated state, in which Li is bonded only to NP but not to electrolyte anion (*N*_ad_ = 1.0, Fig. 3c). Figure 3d shows the total value of *N*_ad_ summed over all Li atoms as a function of time during 0.8 ps after the removal of electrons. The initial and final values of *N*_ad_ were almost the same between Si-free and Si-doped systems. The delithiation is accompanied by the change of the ionic valency (Fig. S2) as expected from Eq. (1), though the numbers of injected and ejected electrons, as well as the time duration of the simulations, are insufficient to observe the complete conversion from Fe^0^ to Fe^3+^, as is inferred from previous X-ray photoelectron spectroscopy (XPS) data^35^. Dissociation of Li ions on the Si-free NP started and the total *N*_ad_ dropped very rapidly immediately after discharge (S1.mov in supplementary information). The dissociation reaction progressed around 80% during 0.05 ps, and almost completed by 0.3 ps. On the other hand, in the Si-doped system, the delithiation reaction proceeds more gently than in the Si-free system (S2.mov in supplementary information). It is conceivable that such a gentle dissociation behavior reduces the mechanical load and damage on electrodes, thereby resulting in improved cyclability.

## Structural stabilization by Si-doping

**Figure 4:**
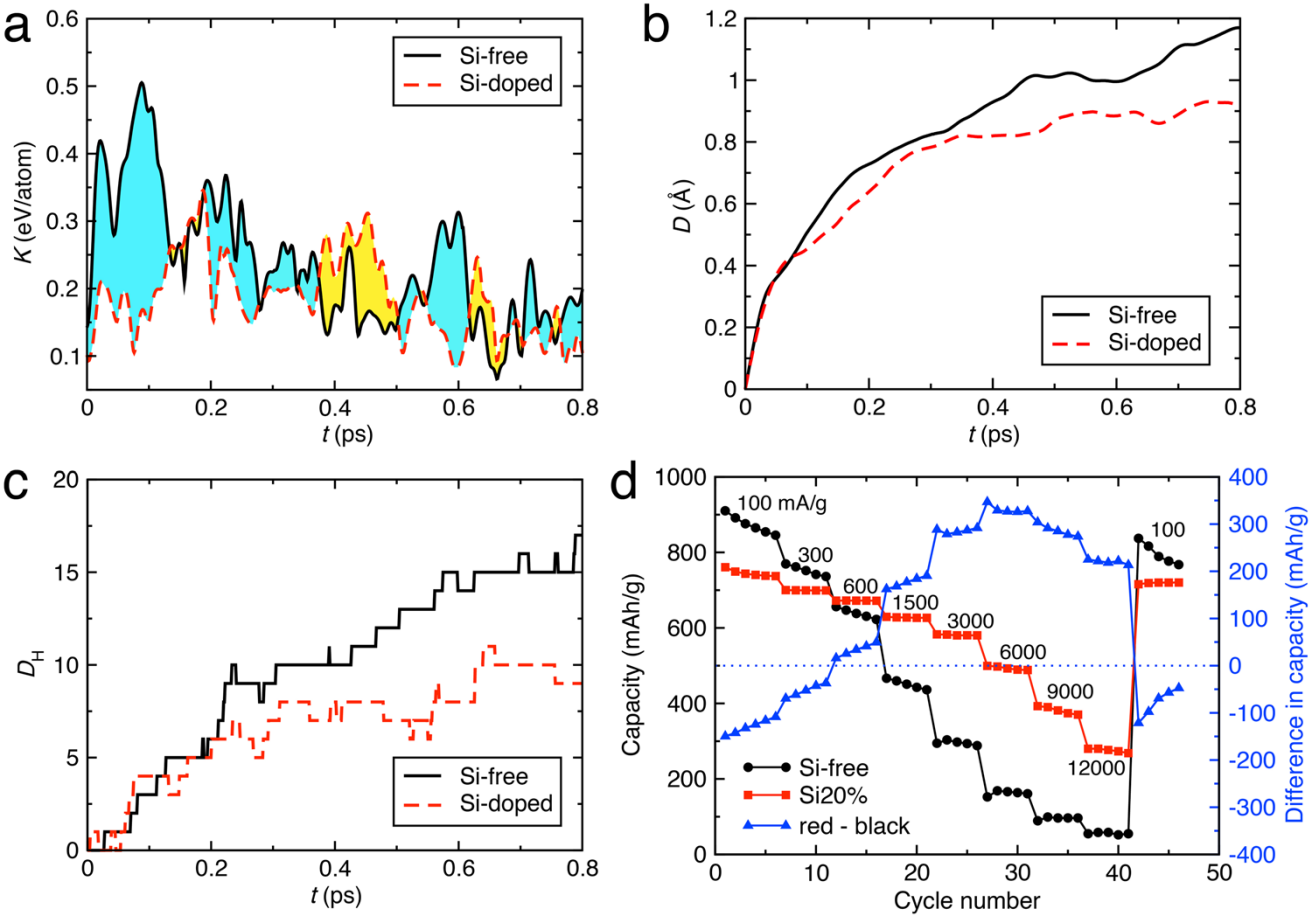
Structural stabilization and improvement of battery rate capability by Si-doping. (**a**–**c**) Time evolution of the average kinetic energy of dissociated Li atoms (**a**) geometrical deformation of NPs (**b**) and the Hamming distances (**c**) of the Si-free (black solid lines) and S-doped (red dashed lines) NPs during the delithiation simulations. (**d**) Experimental measurements of battery rate capabilities for the 2Fh (black), 20% Si-doped 2Fh (red) and their difference (blue with second y axis). In (**a)** cyan and yellow colors indicate the areas where the average kinetic energy for the Si-free system is greater and less than that for the Si-doped system, respectively.

To quantify the structural stability of NPs during the delithiation process, Fig. 4a shows the average kinetic energies of the Li atoms that were dissociated from the NPs at the end of the simulation. Rapid increase of the kinetic energy is observed immediately after the removal of electrons in the Si-free system but not in the Si-doped system. The former may impose large stress on a NP and lead to structural deformations that are detrimental for the structural integrity of electrodes. To quantify geometric deformations of NPs, translationally and rotationally minimized root mean square displacements *D* were calculated by

2 $$\,D={\min }_{b,A}\sqrt{\frac{1}{N}\sum _{i=1}^{N}{({{\bf{r}}}_{i}^{0}-{\bf{b}}-{\bf{A}}{{\bf{r}}}_{i}^{t})}^{2},}$$

where *N*, **r**^*t*^_*i*_, **b** and **A** are the number of atoms in a NP, atomic position of the *i*-th atom at time *t*, translational vector and rotation matrix, respectively^36^. Figure 4b shows reduced *D* for the Si-doped system compared to that of the Si-free system, indicating that the structural stability of NP during delithiation is increased by Si-doping. In order to characterize topological stability, the Hamming distances *D*_H_ of the NPs (Fig. 4c) were calculated to quantify the number of chemical bond-breakage and bond-formation events:

3 $${D}_{H}=\frac{1}{2}\sum _{i,j}^{N}|{\delta }_{i,j}^{0}-{\delta }_{i,j}^{t}|\,,$$

where *δ*^*t*^_*i*,*j*_ = 1 if bond exists between *i*-th and *j*-th atoms at time *t*, and 0 otherwise. Figure 4c shows that the topological change of the Si-doped NP is about 30% lower than that of the Si-free NP. These results show that Si-doping enhances the structural stability of BIOX NP, thereby increasing the cyclability of BIOX-based lithium-ion battery electrodes.

To test the hypothesis that the enhanced structural stability during delithiation of BIOX NPs due to Si-doping leads to improved cyclability of BIOX NP-based batteries, we experimentally measured the rate capability of BIOX-based lithium-ion battery. 2032 coin-type cells using 2-line ferrihydrite (2Fh, also called iron(III) oxide-hydroxides) and 20% Si-doped 2Fh for electrodes were prepared^33^. Here, we used the Si-doped 2Fh as a basis of the primary NPs in the BIOX. The cell performance was evaluated using an electrochemical analyzer (Toscat2000; Toyo System, Japan) under galvanostatic conditions in the voltage range of 0.3–3.0 V at 100–12,000 mA/g. The rate capability of the 2Fh samples were measured at current rates of 100, 300, 600, 1,500, 3,000, 6,000, 9,000, 12,000 mA/g. It was confirmed that the rate capabilities of the Si-20% system are higher than that of the 2Fh system above 600 mA/g and keeps lower fading as the current rate increases (Fig. 4d). This result is consistent with the kinetic, geometrical and topological behaviors in the lithiation/delithiation processes at high charging rate shown above. These values in the electrochemical property are comparable not only to those obtained for anodes with bacteriogenic samples (BIOX) but also to those in previous reports^17–21^ for other nanostructured iron oxides, *e.g*., Table 1 in ref.^21^. Thus, the essential feature of capacity enhancement solely by Si doping is clearly seen in this measurement.

Furthermore, to investigate the medium-range structural change and porosity of the NPs, distribution of cation (Fe and Si)-anion (O) rings in the NPs at delithiated states are analyzed using King’s shortest-path criterion (Fig. S3)^37,38^. In the Si-free NP, the numbers of small four-membered rings and large ten-membered rings were reduced and the average ring size decreased upon lithiation and delithiation cycle, while the Si-doped NP showed opposite trends. It has been shown that the increased ring size is associated with tetrahedrally-coordinated atoms, which is inversely proportional to the number density^39,40^. Therefore, this result indicates that the four-coordinated Si atoms doped in NP reduces structural changes and increases porosity, resulting in enhanced capability and cyclability.

## Chemical origin of Si-dope-induced structural stability

**Figure 5:**
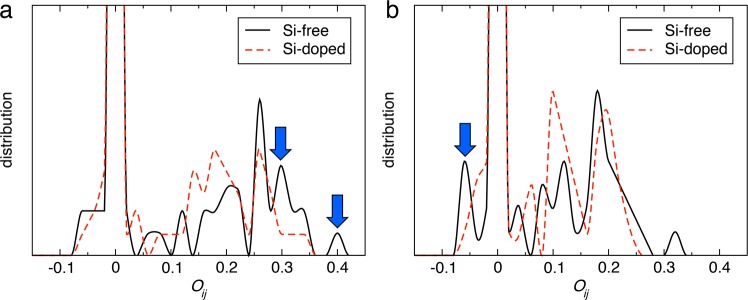
Change of chemical bonding by Si-doping. Distribution of the bond overlap population between Li and O before (**a**) and immediately after (**b**) removing electrons. Black solid and red dashed lines are for the Si-free and Si-doped cases, respectively.

In order to understand the fundamental mechanisms underlying the Si-doping-induced structural stabilization shown above, we estimated the bond strengths by performing bond overlap populations (BOP) analysis^41^. The BOP measures the covalency of chemical bonding. The BOP distributions for Li-O bonds before and immediately after removing electrons are shown in Fig. 5. The sharp peaks (pointed by blue arrows) at large positive bond orders in Fig. 5a indicate that strong bonds exist in the Si-free system. In contrast, the sharp peak (pointed by blue arrow) at a negative bond order in Fig. 5b represents anti-bonding in the Si-free system after removing electrons. Thus, Si-doping reduces the strength of covalent bonds. We also found that not only covalency but also ionicity of Li-O bonds in the Si-doped system is reduced compared to that in the Si-free system (Figs S4 and S5). The weaker chemical bonding in the Si-doped system explains less violent dissociation behaviors observed in the Si-doped system (S2.mov) compared to the Si-free system (S1.mov). Namely, the key structural stabilization mechanism is the weakening of Li-O bond, *i.e*., gentler lithiation and delithiation involving small binding energy with Si doping. Remarkable plastic deformation exhibited by Si at the nanoscale has also been utilized in lithium battery electrodes^42^. Recent reports have shown that nanostructured SiO_2_-based anodes can exhibit high cyclability comparable to the present study, with higher capacity (>1000 mAh/g) than other oxide-base anodes^43–45^. The BIOX-based anode, on the other hand, has its cost effectiveness due to its self-assembled/biogenous manner in its fabrication process.

In summary, our QMD simulations revealed the key role of Si-doping for the structural stability of BIOX NPs during lithiation and delithiation, which leads to the enhanced cyclability of Li-ion batteries as demonstrated by our biomimetic synthesis and characterization. Recent developments in bio-doping technologies have enabled intricate control of the chemical composition of biogenous NPs by simply changing the solutions in which the bacteria are cultivated^46,47^. The fundamental understanding of chemical bonding in BIOX NPs in this work may lead to sustainable synthesis of a wide variety of doped BIOX materials with desired properties.

## Methods

### Simulation methods

In our QMD simulations, the electronic states were calculated using projector augmented-wave method within a framework of spin-polarized density functional theory, in which the generalized gradient approximation was used for the exchange-correlation energy^48,49^. To consider the on-site Coulomb repulsion among the localized electrons, the DFT + *U* method was employed with *U*_eff_ = 4.00 eV for Fe 3*d* electrons^50^. The plane-wave cutoff energies were 25 and 250 Ry for the electronic pseudo-wave functions and pseudo-charge density, respectively. Projector functions were generated for the 3*d*, 4 *s*, and 4*p* states of Fe, 2 *s* and 2*p* states of O, Li and F, and 3 *s*, 3*p* and 3*d* states of P. The local magnetic moments were taken into account for Fe in the antiferro-magnetic alignment. The equations of motion were numerically integrated with a time step of 1.2 fs in a canonical ensemble. A total of 212 and 215 atoms (12Fe_2_O_3_ and 9Fe_2_O_3_-6SiO_2_, respectively, immersed in 19LiPF_6_) in a cubic supercell with a side length of *L* = 18.26 Å was thermalized at 500 K to enhance the mobility of Li ions. The periodic boundary conditions were applied in all directions. The Γ point was used for Brillouin zone sampling for electronic structure calculation. The total simulation time was 7.986 ps. We reproduced the lithiation and delithiation processes by injecting and eliminating electrons for the supercell, respectively. We used a uniform background charge, with a total charge number of *Q* = 0 from simulation time *t* = 0 ps to 1.331 ps, *Q* = 18 from *t* = 1.331 ps to 6.655 ps, and *Q* = 0 again from *t* = 6.655 ps to 7.986 ps. The interatomic distances of F to F in PF_6_^−^ unit structures are constrained from *t* = 1.331 ps to avoid disintegration of PF_6_^−^.

### Experimental methods

The 2Fh sample was prepared by the method of Smith *et al*.^51^, and the Si-doped 2Fh samples were prepared by modifying the Smith’s method in the following manner: First, 20 g of Fe(NO_3_)_3_•9H_2_O (Nacalai Tesque, 99.0%) was crushed to a fine powder by an alumina mortar and pestle and dissolved in 10 mL of 2-propanol (Nacalai Tesque, 99.0%). Subsequently, an appropriate amount of tetraethyl orthosilicate (Nacalai Tesque 95.0%) was added to the solution. The Si concentration, *x* = Si/(Si + Fe), was adjusted to *x* = 0–0.5 in increments of 0.1. Then, about 48 g of NH_4_HCO_3_ (Nacalai Tesque 96.0%) was added to the solution and mixed using an alumina pestle until the rate of CO_2_ generation became slow enough and a thick, red ocher paste formed. The obtained paste was moved to a polytetrafluoroethylene beaker and left for 6–12 h at room temperature to complete the reaction. Finally, the paste was washed with ~2 L of distilled water and ~200 mL of ethanol and dried under vacuum for 2 days at room temperature.

## Supplementary information

Suuplementary information

S1.mov

S2.mov
