# Supplementary material for: Rapid and reversible lithiation of doped biogenous iron oxide nanoparticles
Source: Sci Rep. 2019 Feb 12;9:1828. doi: 10.1038/s41598-019-38540-8 (PMC6372689; doi:10.1038/s41598-019-38540-8)
Supplement: Supplementary file 1 — Suuplementary information [file 41598_2019_38540_MOESM1_ESM.pdf]

SUPPLEMENTARY INFORMATION

Rapid and reversible lithiation of doped biogenous iron oxide nanoparticles

Masaaki Misawa,[1,2,3] Hideki Hashimoto,[4] Rajiv K. Kalia,[3] Syuji Matsumoto,[5,6] Aiichiro Nakano,[3*] Fuyuki Shimojo,[2] Jun Takada,[5,6] Subodh Tiwari,[3] Kenji Tsuruta,[6] and Priya Vashishta[3]

[1]Faculty of Science and Engineering, Kyushu Sangyo University, Fukuoka 813-8503, Japan.
[2]Department of Physics, Kumamoto University, Kumamoto 860-8555, Japan.
[3]Collaboratory for Advanced Computing and Simulations, Department of Physics & Astronomy, Department of Computer Science, Department of Chemical Engineering & Materials Science, and Department of Biological Sciences, University of Southern California,
Los Angeles, CA 90089-0242, USA.
[4]Department of Applied Chemistry, School of Advanced Engineering, Kogakuin University, Tokyo 192-0015, Japan.
[5]Core Research for Evolutionary Science and Technology (CREST), Japan Science and Technology Agency (JST), Okayama University, Okayama, 700-8530, Japan.
[6]Graduate School of Natural Science and Technology, Okayama University, Okayama 700-8530, Japan.
*Corresponding author. Email: anakano@usc.edu

Overview.

Description of videos S1.mov and S2.mov

Figures S1-S5

Videos.

QuickTime movie, S1.mov and S2.mov, show rapid delithiation of Fe_2O_3 and Fe_2O_3-SiO_2 nanoparticles (NPs), respectively. The green spheres represent Li atoms and the other atoms in the nanoparticles are shown by gray. Eighteen electrons were removed at t = 0 ps.

Results.

X-ray diffraction (XRD) patterns of Si-2Fh were observed at room temperature using a Rigaku RINT-2000 diffractometer with Cu Ka radiation. Diffraction intensities of the samples were measured by normal Θ-2Θ scan with a scan step of 0.02°.

Two broad diffraction peaks around 2Θ = 35° and 63° corresponding to the (11l) and (30l) planes of 2Fh,[1] respectively, reveal the formation of a monophasic 2Fh for all the samples, as shown in the XRD patterns (Fig. S1). The peaks around 2Θ = 35° shows a lattice constant of d = 2.7 Å. These two peaks are gradually broadened and shifted to lower angles with the increasing Si molar ratio until x = 0.30, revealing that the loaded Si is incorporated into the structure of iron oxyhydroxide. For x = 0.40, the first broad peak around 2Θ = 35° split into two peaks, suggesting local elemental inhomogeneity: The samples could contain Fe- and Si-rich regions.

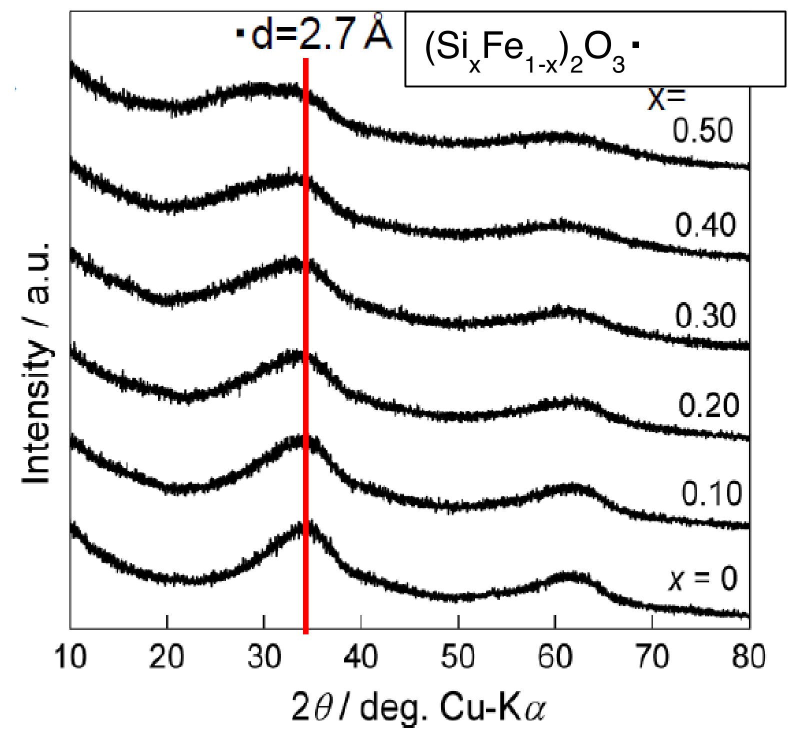

Figure S1 | XRD pattern of Si-2Fh. Experimental XRD patterns of 2Fh (x = 0) and Si-doped 2Fh with different compositions (x = 0.10-0.50).

To investigate ionicity of atoms, atomic net charges were calculated based on population analysis. The atomic net charge of Fe before, immediately after and 1.8 ps after removing electrons are shown in Fig. S2. In the charged state, nearly all Fe atoms have charge in the range of 0.5 to 0.8, indicative of the Fe^{2+} state. After removing electrons, Fe charges became more positive and eventually reached to around 1.1, corresponding to the conversion of Fe^{2+} to Fe^{3+}. The atomic charges of Li and Si atoms were also calculated.

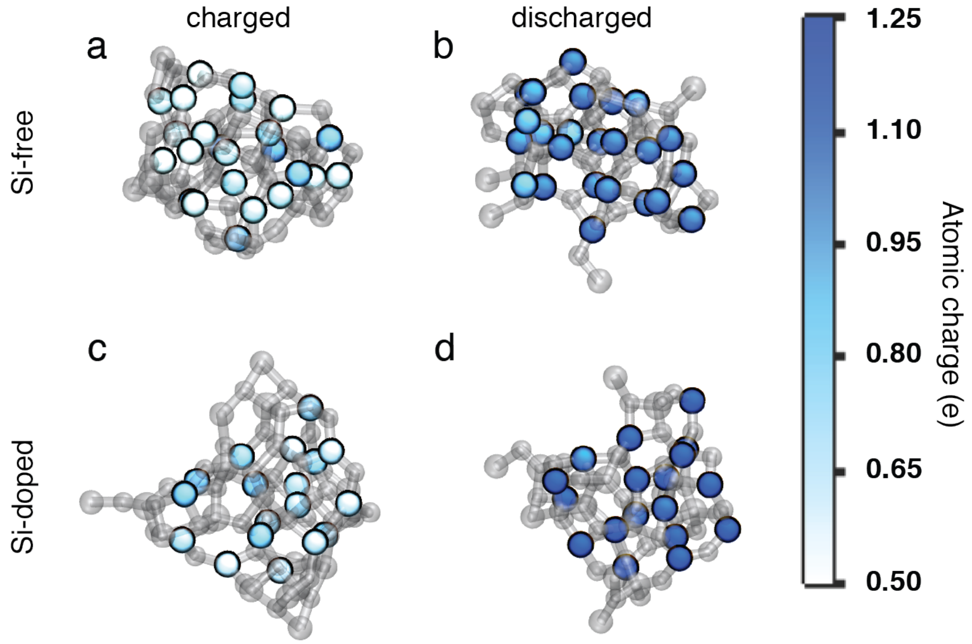

Figure S2 | Atomic charges of Fe atoms. Atomic charges of Fe atoms in (**a, b**) Fe_2O_3 and (**c, d**) Fe_2O_3-SiO_2 NPs; (**a**, **c**) are before removing electrons, whereas (**c**, **d**) are after delithiation reactions. Opaque spheres represent Fe atoms. The charge of Fe atoms is color coded.

Figure S3 shows the distribution of cation (Fe,Si)-anion (O) rings in the NPs at delithiation states. The ring statistics was analyzed by King's shortest path criterion.[2,3] In the Si-free NP, the ring distribution changed drastically upon the lithiation and delithiation cycle and the average ring size decreased. On the other hand, in the Si-doped NP, the number of small (four or six-membered) rings was completely preserved. Additionally, large twelve-membered rings are generated only in the Si-doped NP after first delithiation.

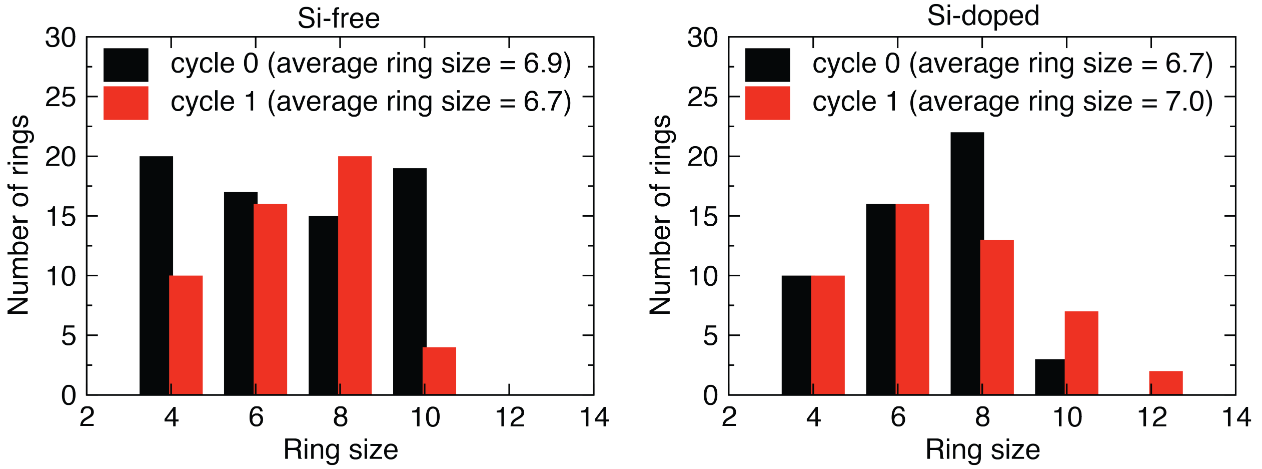

Figure S3 I Ring statistics. Distributions of cation (Fe and Si)-anion (O) rings in the Si-free (left) and Si-doped (right) NPs before the injection of electrons (black) and after first delithiation (red).

Figure S4, a-f, shows Li charges in Si-free (a-c) and Si-doped (d-f) systems, whereas Si charges are shown in Fig. S3, g-i. These charges did not change appreciably by removing electrons, in contrast to Fe charges.

Figure S5 shows the atomic net charges of O atoms. We can see that the O charges in the Si-free system are more negative than that in the Si-doped system. This negativity causes the strong ionicity of Li-O bonds in the Si-free system.

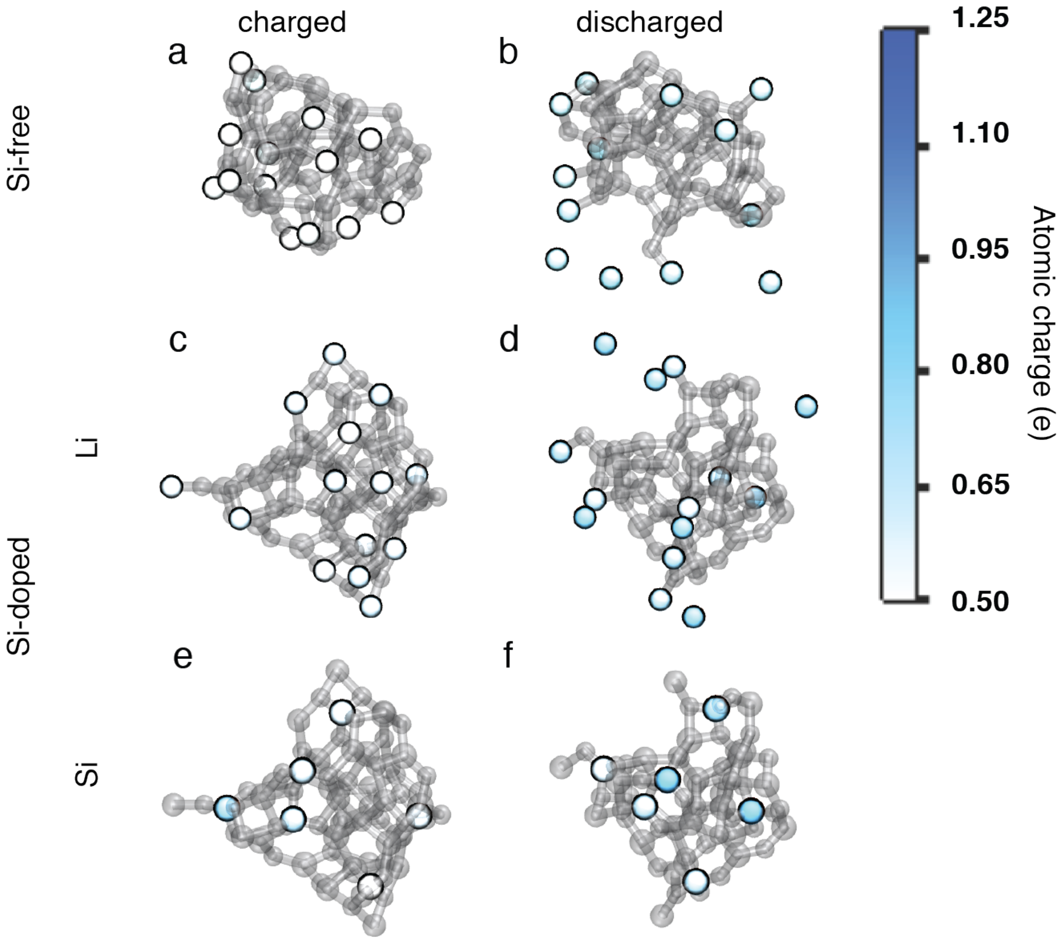

Figure S4 | Atomic charges of Li and S atoms. Atomic charges of Li (**a-d**) and Si (**e, f**) atoms in (**a, b**) Fe_2O_3 and (**c-f**) Fe_2O_3-SiO_2 NPs; (**a**, **c, e**) are before removing electrons, whereas (**b, d, f**) after delithiation reactions. Opaque spheres represent Li and Si atoms. The charge of Li and SI atoms is color coded.

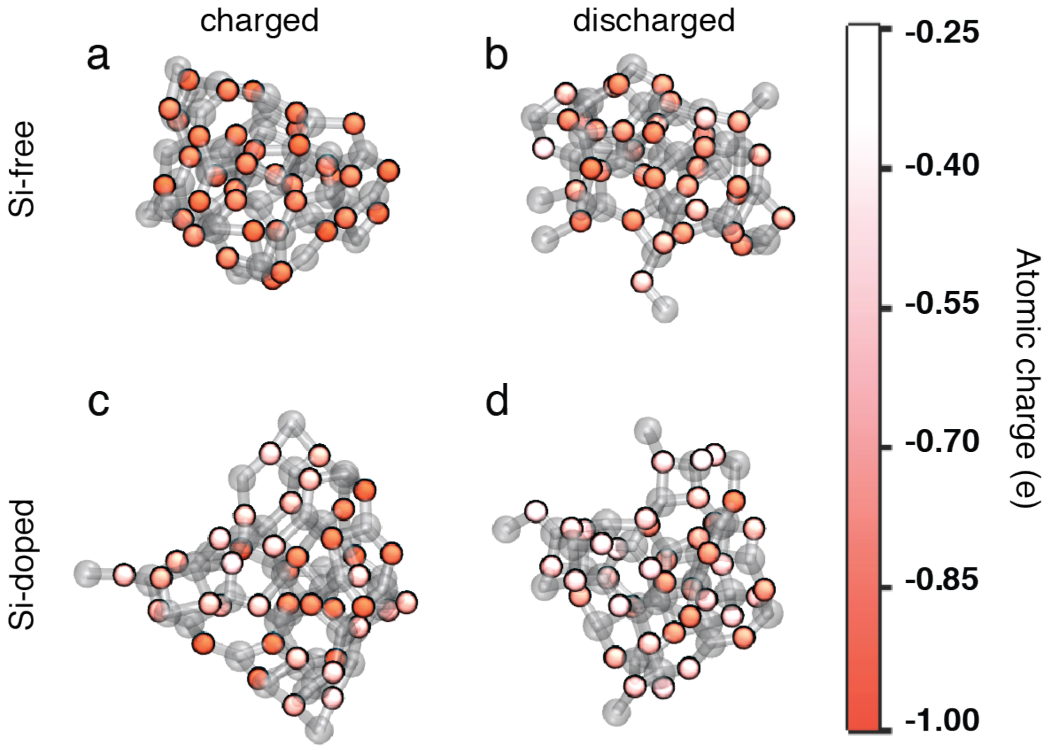

Figure S5 | Atomic charges of O atoms. Atomic charges of O atoms in (**a, b**) Fe_2O_3 and (**c, d**) Fe_2O_3-SiO_2 NPs; (**a**, **c**) are before removing electrons, whereas (**b**, **d**) are after delithiation reactions. Opaque spheres represent O atoms. The charge of O atoms is color coded.

Reference for Supplementary Information.

1 Eggleton, R. A. & Fitzpatrick, R. W. New data and a revised structural model for ferrihydrite. *Clay Clay Miner* **36**, 111-124, doi:Doi 10.1346/Ccmn.1988.0360203 (1988).

2 Franzblau, D. S. Computation of ring statistics for network models of solids. *Physical Review B* **44**, 4925, doi:10.1103/PhysRevB.44.4925 (1991).

3 King, S. V. Ring Configurations in a random network model of vitreous silica. *Nature* **213**, 1112-1113, doi:10.1038/2131112a0 (1967).
